# Hierarchy in the home cage affects behaviour and gene expression in group-housed C57BL/6 male mice

**DOI:** 10.1038/s41598-017-07233-5

**Published:** 2017-08-01

**Authors:** Yasuyuki Horii, Tatsuhiro Nagasawa, Hiroyuki Sakakibara, Aki Takahashi, Akira Tanave, Yuki Matsumoto, Hiromichi Nagayama, Kazuto Yoshimi, Michiko T. Yasuda, Kayoko Shimoi, Tsuyoshi Koide

**Affiliations:** 1 Mouse Genomics Resource Laboratory, National Institute of Genetics, Yata, Mishima, Shizuoka, 411-8540 Japan; 2 Laboratory of Biochemistry and Toxicology, Graduate School of Integrated Pharmaceutical and Nutritional Sciences, University of Shizuoka, Yada, Shizuoka 422-8526 Japan; 3 Faculty of Agriculture, University of Miyazaki, Gakuen Kibanadai Nishi, Miyazaki, 889-2192 Japan; 4 Laboratory of Behavioral Neuroendocrinology, University of Tsukuba, Tsukuba, 305-8577 Japan; 5 Department of Genetics, SOKENDAI, Yata, Mishima, Shizuoka, 411-8540 Japan

## Abstract

Group-housed male mice exhibit aggressive behaviour towards their cage mates and form a social hierarchy. Here, we describe how social hierarchy in standard group-housed conditions affects behaviour and gene expression in male mice. Four male C57BL/6 mice were kept in each cage used in the study, and the social hierarchy was determined from observation of video recordings of aggressive behaviour. After formation of a social hierarchy, the behaviour and hippocampal gene expression were analysed in the mice. Higher anxiety- and depression-like behaviours and elevated gene expression of hypothalamic corticotropin-releasing hormone and hippocampal serotonin receptor subtypes were observed in subordinate mice compared with those of dominant mice. These differences were alleviated by orally administering fluoxetine, which is an antidepressant of the selective serotonin reuptake inhibitor class. We concluded that hierarchy in the home cage affects behaviour and gene expression in male mice, resulting in anxiety- and depression-like behaviours being regulated differently in dominant and subordinate mice.

## Introduction

Mice are useful model animals for studying a wide range of human diseases, including psychological disorders. A variety of behavioural tests are widely used to assess higher-order brain functions. Behaviour is strongly affected by the experimental conditions used^1–4^, so it is crucial that, as far as possible, experiments are performed using mice with the same genetic background and sex, and under identical environmental conditions. Males are preferred over females for use in behavioural studies because males do not experience dynamic hormonal changes associated with the oestrous cycle^5^. However, the behaviour of males still varies widely under some experimental conditions. This was the case even when genetically identical mice from an inbred strain were kept under the same environmental conditions in behavioural experiments^5^.

It is recommended in many animal care and ethical guidelines that mice should be kept under group housing to decrease the stress caused by social isolation^5, 6^. These recommendations are largely based on previous reports of male mice housed individually exhibiting behavioural differences from those housed in groups in terms of aggression, locomotor activity, immobility in forced swim tests, novel object recognition, fear conditioning, stereotypies, convulsions, nervousness, tractability, and other behaviours^7–10^. However, confinement of several adult male mice to a small space may be unnatural because male mice are territorial and fend off other males to maintain an individual’s territory^11^. Mice are social animals, but male mice are aggressive to conspecific males once they attain sexual maturity^11, 12^; such behaviour is natural and adaptive for a territorial animal. Housing groups of male mice together can lead to aggressive behaviour towards cage mates and frequent injury in some mouse strains^13–15^. Aggressive behaviour in males caged together largely depends on the genetic background of the mouse strain used, but agonistic behaviour between males in a cage has been observed in a wide range of mouse strains.

Under experimental conditions, agonistic interactions between male mice affect various types of behaviour, and physiological and molecular phenotypes. Male mice exposed to social defeat stress show depression-like phenotypes compared with non-defeated control males^16^. Dominant animals have different synaptic efficacies to subordinate animals^17^. Characteristic differences in gene expression in dominant and subordinate animals are detectable. Dominant animals show higher expression of oestrogen receptor alpha, corticotropin releasing hormone receptor 2, and androgen receptors in the nucleus accumbens and bed nucleus of the stria terminalis^5^, which regulate social behaviour and behavioural responses to stress^18^. Brain-derived neurotrophic factor (BDNF), which is related to neural cell functions, including adult neurogenesis, is expressed more highly in the hippocampi of dominant males than in the hippocampi of subordinate males^19^. These previous findings suggest that agonistic interactions between male mice alter neurological function in individuals kept under social conditions.

Social hierarchy is a fundamental survival strategy in most animals and provides dominant animals with advantages in terms of access to vital resources without intense, wasteful, and fatal fights occurring between group members. Social hierarchy also gives subordinates the benefit of avoiding intense and fatal fights, and provides a safe social environment^20^. Social hierarchy is stable once established^17^ and subordinates are attacked by a dominant male each day.

The main objective of the present study was to determine whether the behaviours and other phenotypes of male mice housed together were affected by agonistic interactions between the mice that occurred to establish and maintain a social hierarchy that naturally occurs in mice in group housing.

## Results

### Analysis of social hierarchy in group-housed male mice

The social hierarchy in mice in their home cage was determined by observing agonistic behaviour (Table 1) in video images recorded immediately after bedding was changed and/or the lighting was switched off at the start of a period of darkness (Movie S1). The average frequency of agonistic behaviour in each period over 4 sampling days was used to determine the social rank of each mouse.

**Table 1:** Definition of agonistic behaviours.

Offensive behaviour	Aggressive action	Aggressive grooming and chasing an opponent
	Submission induction	Eliciting defensive uprighting (submissive) behaviour to an opponent
Defensive behaviour	Aggression received	Receiving aggressive grooming and being chased by an opponent
	Submissive reaction	Defensive uprighting (submissive) behaviour to an opponent

**Figure 1:**
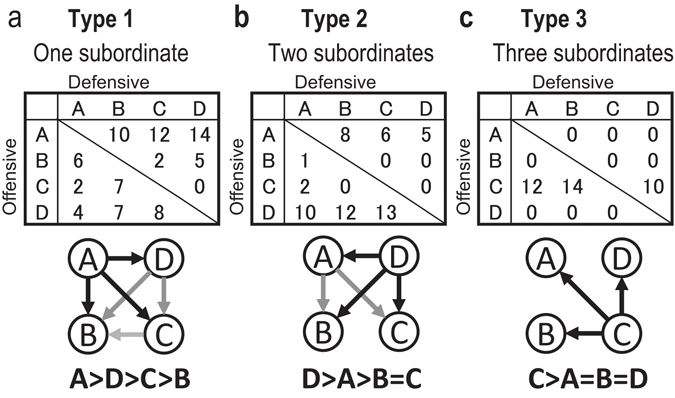
Determination of the social hierarchy in a group-housing cage. The social hierarchy in a home cage was determined from the home-cage dyadic agonistic interactions. The mouse that exhibited offensive behaviour and elicited submissiveness from all other mice was designated the dominant mouse. Mice that exhibited submissive behaviour to other mice but never dominated another cage mate were designated subordinate. (**a**) Type 1: a linear hierarchy was estimated when the relationships between individuals were transitive (i.e., if mouse A dominated mice B, C and D, mouse D dominated mice B and C, and mouse C dominated mouse B). In this manner, the dominant and subordinate males were defined as the individuals with the highest and lowest ranking, respectively, and the mice intermediate between the dominant and subordinate males were considered to be intermediate males. (**b**) Type 2: two subordinates were identified when no agonistic interactions were observed between the two lowest ranking mice. (**c**) Type 3: a case with one dominant and three subordinate mice.

As shown in Fig. 1, the social ranks of the mice in the home cage were divided into three groups, namely dominant, subordinate, and the other mice that showed intermediate behaviour. We first determined whether each mouse was a ‘winner’ or a ‘loser’ relative to each other mouse in the cage. A winner displayed offensive behaviour (aggressive grooming and chasing) more often and defensive behaviour (flight and taking a defensive upright position that indicates submissive behaviour) rarely, and the losers followed the opposite pattern^21, 22^. The dominant individual in the cage (the mouse that won in confrontations with all of the other mice in the cage) as well as the most submissive mice were identified. The other intermediate mice were defined as those exhibiting offensive behaviour often but were attacked by other mice. In this study, we only focused on the dominant and subordinate individuals and excluded the intermediate males from analyses.

Three hierarchical structures were observed in the cages that we analysed. These were: Type 1, one dominant mouse and one subordinate mouse (Fig. 1a); Type 2, one dominant mouse and two subordinate mice (Fig. 1b); and Type 3, one dominant mouse and three subordinate mice^21^ (Fig. 1c). We observed 40 cages in three experiments, and observed Type 1, 2 and 3 hierarchical structures in 13, 9 and 15 cages, respectively. No social hierarchy was identified in three of the 40 cages because agonistic interactions occurred infrequently or the hierarchy was non-linear (see the Methods section for details).

### Relationships between agonistic behaviour and physical growth and general behaviour

**Figure 2:**
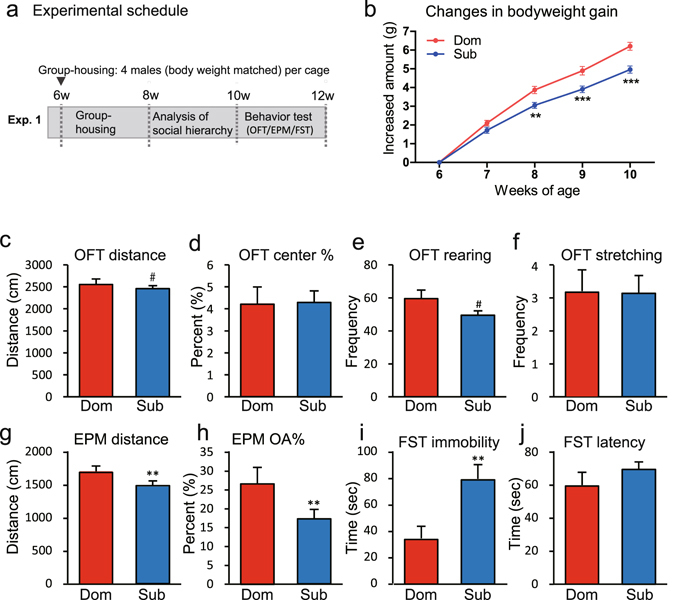
Effects of social hierarchy on growth and behaviour. (**a**) Experimental schedule. The social hierarchy was determined by analysing dyadic agonistic interactions between each combination of two males in the home cage for 2 weeks (when the mice were aged 8–10 weeks). In Experiment 1, all of the animals were used in behavioural tests at age 10–12 weeks (*n* = 16 cages). (**b**) Comparison of body weight gains in different hierarchies in group-housed mice (*P < 0.05, **P < 0.01, dominant versus subordinate). The behaviours of the dominant (Dom) and subordinate (Sub) males were compared. (**c**–**f**) Open-field test (OFT) results for (**c**) the distance travelled, (**d**) the percentage of time spent in the central area, (**e**) the rearing frequency, and (**f**) the stretching frequency. (**g** and **h**) Results of the elevated plus-maze (EPM) test, showing (**g**) the distance travelled and (**h**) the percentage of time spent in the open arms. (**i** and **j**) Results of the forced swim test (FST), showing (**i**) the immobility time and (**j**) latency to immobility (^#^ *P* < 0.1, **P < 0.01).

We investigated the effects of social rank on physical growth and anxiety- and depression-like behaviours (Fig. 2a,b). The two-way repeated measure ANOVA results showed a significant main effect of social rank (*F*[1,248] = 7.75, *P* < 0.001) as well as a significant interaction (*F*[4,248] = 10.53, *P* < 0.0001) on body weight gain. A *post-hoc* test showed that the body weights of the subordinate males increased significantly less than the body weights of the dominant males at 8, 9 and 10 weeks of age (Fig. 2b).

We investigated the effects of social hierarchy on anxiety- and depression-like behaviours by conducting a series of behavioural tests using the group-housed mice. In open-field tests, the travelling distance (*w* = 68.0, *P* < 0.1) and the frequency of rearing (*w* = 76.0, *P* < 0.1) tended to be lower in subordinate than in dominant male mice (Fig. 2c,e). In elevated plus-maze tests, subordinate males showed significantly lower travelling distances (*w* = 81.0, *P* < 0.01) and percentage of time spent in the open arm (*w* = 81.0, *P* < 0.01) than did dominant males (Fig. 2g,h). In forced swim tests, subordinate males showed significantly longer duration of immobility (*w* = −96.0, *P* < 0.05) than that of dominant males (Fig. 2i). These behavioural test data suggested that subordinate mice showed more anxiety- and depression-like behaviour than dominant mice.

### Comparison of stress levels in dominant and subordinate males

**Figure 3:**
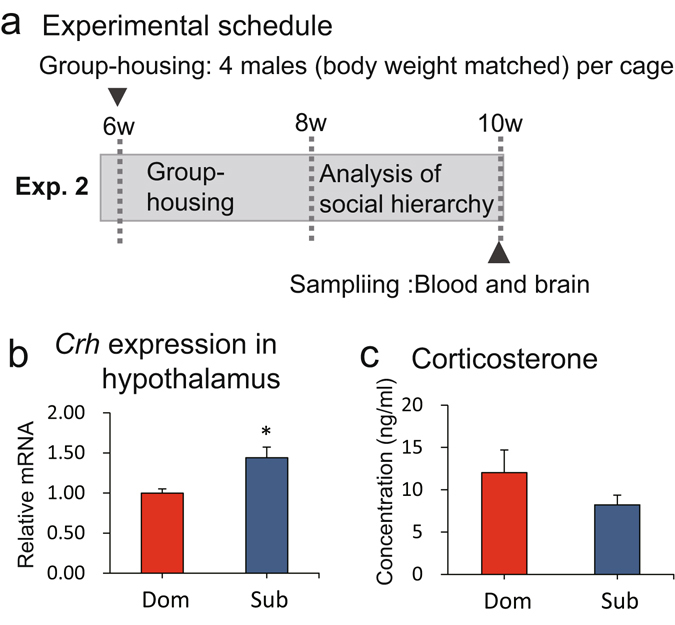
Comparison of stress levels in dominant and subordinate males. The hypothalamic expression of *Crh* and serum concentrations of corticosterone in dominant (Dom) and subordinate (Sub) males were compared. (**a**) In Experiment 2, blood samples and the brain were collected when the mice were aged 10 weeks, and the hormone concentrations and mRNA transcript levels were determined. Blood (*n* = 16 cages) and brains were collected from different sets of animals (*n* = 16 cages and *n* = 8 cages, respectively). (**b**) The *Crh* mRNA transcript level in the hypothalamus (*P < 0.05). (**c**) Concentrations of corticosterone in serum, measured to assess basal stress levels in the mice in their home cages.

The stress levels in dominant and subordinate male mice were assessed by comparing expression of the corticotropin-releasing hormone (*Crh*) gene in the hypothalamus and the concentrations of corticosterone in the serum (Fig. 3). The *Crh* mRNA concentration was significantly higher in subordinate than dominant males (*w* = −26.0, *P* < 0.05) (Fig. 3b), whereas the corticosterone concentration in the serum samples from subordinate and dominant males was not significantly different (*w* = 36.0, *P* > 0.1) (Fig. 3c). These results suggested that the subordinate males tended to be more severely stressed in the home cage.

### Comparison of expression of genes associated with anxiety- and depression-like behaviours and adult neurogenesis in dominant and subordinate males

**Figure 4:**
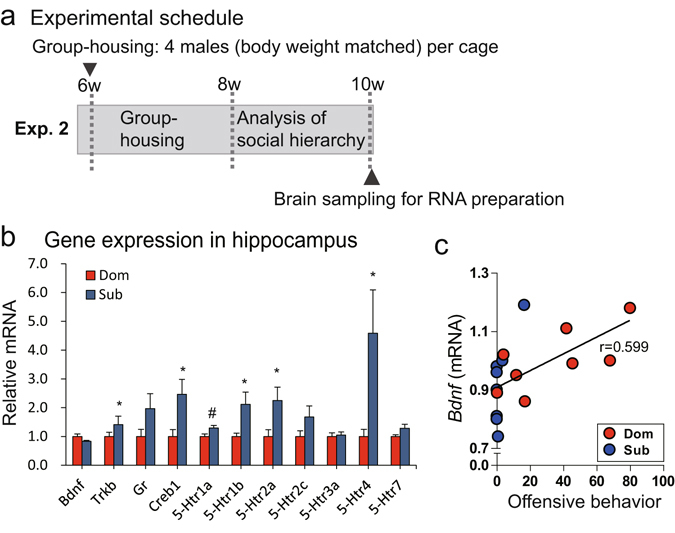
Comparison of gene expression levels in the hippocampi of dominant and subordinate males. (**a**) Experimental schedule. Brains were collected when the mice were aged 10 weeks. (**b**) Expression of genes associated with anxiety- and depression-like behaviour and adult neurogenesis in the hippocampi of dominant (Dom) and subordinate (Sub) males (^#^ *P* < 0.1, *P < 0.05). (**c**) Correlation between *Bdnf* expression and aggressive behaviour frequency.

Neural plasticity and neurogenesis are regulated by BDNF, the synthesis of which is regulated by the phosphorylated cAMP responsive element binding protein, serotonin (5-HT), glucocorticoid and BDNF itself^16, 23, 24^. Serotonin has a range of roles regulating aggression and anxiety- and depression-like behaviours. Previous studies have reported that chronic stress causes down-regulation of *Bdnf* and increases expression of the BDNF receptor gene, *Trkb*, in the hippocampus^16, 23^. It has also been reported that chronic stress affects the 5-HT system in the hippocampus^25^. To examine whether social hierarchy under the group-housing condition alters expression of these genes, we analysed gene expression in the hippocampus. We measured mRNA amounts for *Bdnf*, *Trkb*, the cAMP responsive element binding protein gene *Creb1*, the glucocorticoid receptor gene *Gr*, and several 5-HT receptor subtype genes (*5-Htr1a*, *5-Htr1b*, *5-Htr2a*, *5-Htr2c*, *5-Htr3a*, *5-Htr4* and *5-Htr7*) in the hippocampus (Fig. 4a,b). No significant difference in *Bdnf* mRNA expression was found between dominant and subordinate mice (Fig. 4b), but *Bdnf* mRNA expression was significantly positively correlated with the frequency of offensive behaviour (*P* < 0.05, *r* = 0.599) (Fig. 4c and Table S1). The mRNA transcript levels were higher in subordinate animals than dominant males for *Trkb* (*w* = −24.0, *P* < 0.05)*, Creb1* (*w* = −26.0, *P* < 0.05)*, 5-Htr1b* (*w* = −26.0, *P* < 0.05)*, 5-Htr2a* (*w* = −30.0, *P* < 0.05) and *5-Htr4* (*w* = −34.0, *P* < 0.05) mRNA (Fig. 4b). No difference in *Gr* mRNA transcription was detected between dominant and subordinate males (Fig. 4b). These results suggested that dominant and subordinate male mice show differences in neural plasticity or in the 5-HT system in the central nervous system.

### Comparison of adult neurogenesis in the dentate gyrus in dominant and subordinate males

**Figure 5:**
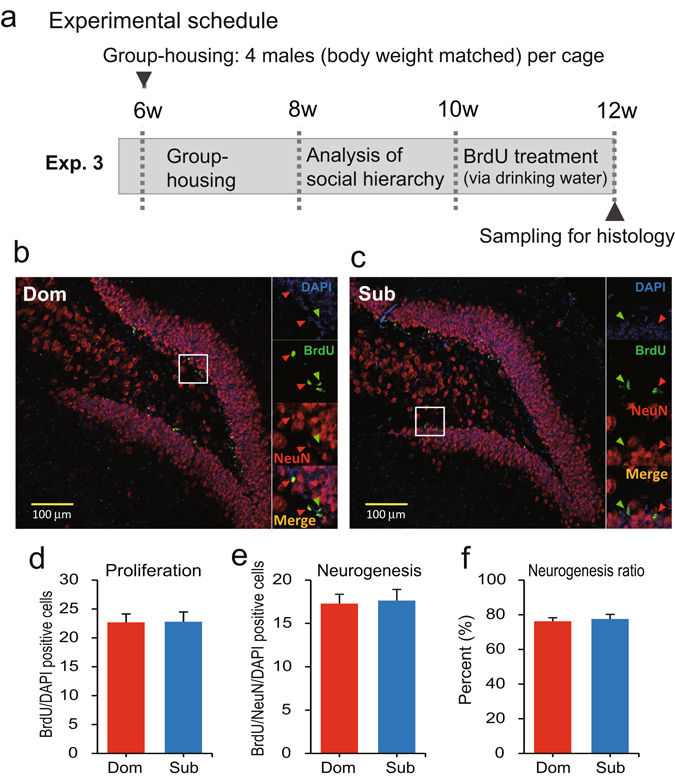
Comparison of adult neurogenesis between dominant and subordinate males in the hippocampus. Neurogenesis analysis was performed on dominant (Dom) and subordinate (Sub) male mice. (**a**) Experimental schedule. BrdU in drinking water (1.2 mg/mL) was supplied when the mice were aged 10–12 weeks, and the brains of the mice were collected when the mice were aged 12 weeks (*n* = 8 cages) for immunohistochemical analysis. Neurogenesis analysis was performed using antibodies against BrdU, and the neural cells were labelled with NeuN antibodies. DAPI was used to detect nuclei. Examples of the immunohistochemistry of samples from a (**b**) dominant and (**c**) subordinate mouse are shown. (**d**–**f**) Comparisons of (**d**) cell proliferation, (**e**) neurogenesis and (**f**) percentage of neurogenesis in newly formed cells in dominant and subordinate mice.

Decreased neurogenesis is one of the major pathophysiological signs of anxiety disorder and depression, therefore we compared hippocampal adult neurogenesis in dominant and subordinate male mice (Fig. 5a–c). No significant differences were observed among measures of neurogenesis in the dentate gyrus (DG) of the hippocampus in the dominant and subordinate males (Fig. 5d–f). These results suggested that the levels of stress experienced by subordinate males because of their social hierarchy ranks were not sufficient to suppress adult neurogenesis.

### Effects of antidepressant treatment on group-housed mice

**Figure 6:**
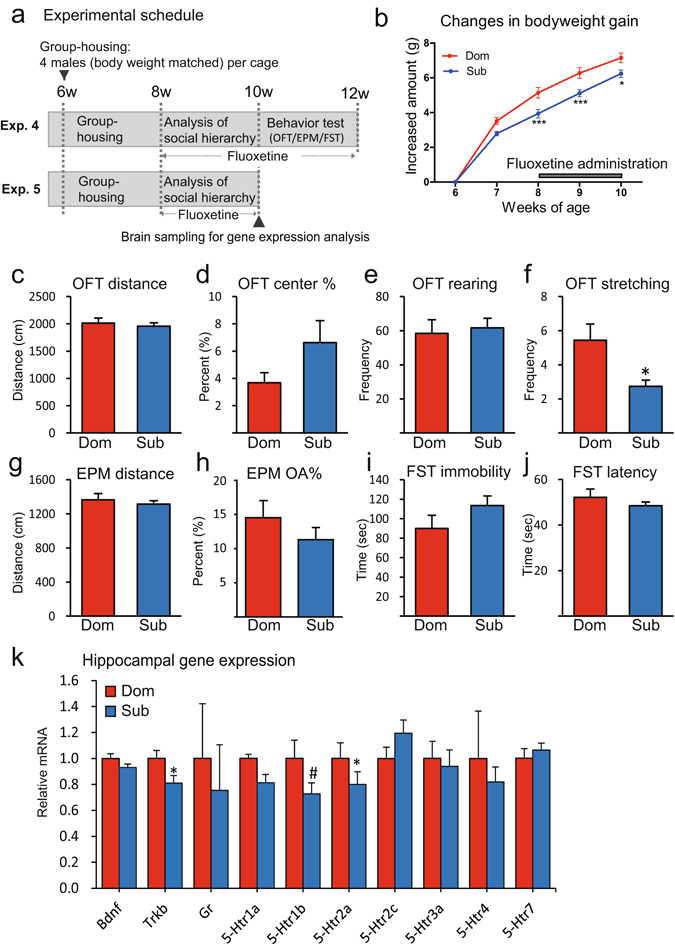
Effects of fluoxetine on behaviour and gene expression. The effects of an antidepressant on anxiety- and depression-like behaviour were assessed by treating group-housed mice with fluoxetine, a selective serotonin reuptake inhibitor, in drinking water. (**a**) Fluoxetine treatment experimental schedule for the behavioural tests and gene expression analysis. In Experiment 4, all animals were used in behavioural tests when they were aged 10–12 weeks (*n* = 16 cages). In Experiment 5, brains were collected when the mice were 10 weeks old, and gene expression analysis was performed (*n* = 8 cages). (**b**) Comparison of body weight gains in group-housed mice with different hierarchical positions (*P < 0.05, **P < 0.01, ***P < 0.001, dominant versus subordinate). (**c**–**j**) Comparison of behaviours in dominant (Dom) and subordinate (Sub) males. (**c**–**f**) Results of the open-field tests (OFT), (**g** and **h**) results of the elevated plus-maze (EPM) tests, and (**i** and **j**) results of the forced swim tests (FST) (*P < 0.05). (**k**) Comparison of gene expression levels in the hippocampi of the Dom and Sub males (^#^ *P* < 0.1, *P < 0.05).

The effects of an antidepressant on anxiety- and depression-like behaviour in mice were investigated by orally treating group-housed mice in their home cages with fluoxetine, which is a selective serotonin reuptake inhibitor (Fig. 6a). In this experiment, we examined whether fluoxetine treatment affected relative differences in phenotype between dominant and subordinate males.

No difference between dominant and subordinate males in duration and frequency of drinking normal water was observed (Fig. S1a,b). In mice treated with fluoxetine, no difference was observed between the amounts of fluoxetine-containing water and normal water consumed (Fig. S1c). Hierarchy formation was still observed in the group of mice treated with fluoxetine. Two-way repeated measure ANOVA showed a significant main effect of social rank (*F*[1,184] = 10.38, *P* < 0.01) and a significant interaction (*F*[4,184] = 6.12, *P* < 0.0001) on body weight gain. A *post-hoc* test revealed that the body weights of subordinate males increased significantly less than the body weights of the dominant males at 8, 9 and 10 weeks of age (Fig. 6b). By contrast, no significant differences were observed between dominant and subordinate males in the distance travelled, percentage of time spent in the centre arena, and rearing in the open-field tests (Fig. 6c–e), the distance travelled and percentage of time spent in the open arms in the elevated plus-maze tests (Fig. 6g,h), and the duration of immobility in the forced swim tests (Fig. 6i). In addition, significantly less stretching behaviour was observed in subordinate than dominant males in open-field tests (*w* = 88.0, *P* < 0.05) (Fig. 6f). These results suggested that fluoxetine treatment abolished differential effects of hierarchy on dominant and subordinate animals under the group-housed condition.

The effects of fluoxetine at the molecular level were investigated by examining gene expression (Fig. 6a Experiment 5, Fig. 6k). Fluoxetine treatment negated or inverted the relative differences in gene expression in dominant and subordinate males that were observed in Experiment 2 (i.e., no fluoxetine administered) (Fig. 6k). Subordinate males showed decreased amounts of *Trkb*, *5Htr1b* and *5-Htr2a* mRNAs compared with dominant males (*Trkb*: *w* = 25.0, *p* < 0.01; *5Htr1b*: *w* = 24.0, *p* < 0.1; *5Htr2a*: *w* = 28.0, *p* < 0.01). No differences in transcription of the other genes analysed were observed between subordinate and dominant males in the fluoxetine treatment (Fig. 6k). The observed changes in relative expression patterns of 5-HT-related genes between dominant and subordinate males after fluoxetine treatment suggested that the serotonergic system in the mice was altered by fluoxetine treatment, which was accompanied by alterations in anxiety- and depression-like behaviours.

## Discussion

Social hierarchy allows dominant mice to obtain preferential access to vital resources, such as food, territory and receptive females, and therefore subordinate animals frequently lose opportunities to access these resources and suffer stress^26^. In this study, we observed that hierarchy in a home cage causes differences in physical growth, behaviour and gene expression in the brains of different individual mice. The subordinate male mice showed increased anxiety- and depression-like behaviours and different amounts of mRNA transcripts of *Crh* and serotonin-related genes compared with those of the dominant male mice.

Chronic social psychological stress is a major risk factor in the development of stress-related mental disorders such as depression and anxiety disorders in humans. An increased basal glucocorticoid concentration caused by chronic stress suppresses neural activity and causes adult neurogenesis in the hippocampus to become dysfunctional^16, 23^, and may result in anxiety disorders and depression^27^. This pathophysiology of social defeat stress has also been observed in rodent models^16, 28^.

We observed that *Crh* mRNA in the hypothalamus, which plays a key role in hypothalamic–pituitary–adrenal axis function, was more highly expressed in subordinate males than in dominant males. However, the serum corticosterone concentration did not differ between dominant and subordinate males. This inconsistency might be because the stress caused by co-housing was relatively mild. Similar inconsistency between *Crh* mRNA amounts and blood corticosterone concentration was reported previously for male rats under chronic mild stress^29^.

Dominant–subordinate relationships may cause a wide range of differences in several physiological functions. It has recently been reported that aggressive dominant male mice kept in a group of 12 males in a large arena showed higher amounts of *Bdnf* mRNA in the hippocampus than those of the other mice in the group^19^. Higher levels of adult neurogenesis in the hippocampus have been observed in territorially dominant mice kept in a large group of mice (*n* = 40) in a large arena compared with those of the other mice in the group^30^. These results indicate that a dominant hierarchical status may alter hippocampal adult neurogenesis, mediating anxiety- and depression-like behaviour. However, we did not detect significant differences in adult neurogenesis or *Bdnf* transcription in the hippocampus of dominant and subordinate males in the present study. The amount of *Bdnf* transcripts was positively correlated with the frequency of offensive behaviour, therefore the differences in the results for subordinate mice between the present and previous studies may have been caused by the subordinate mice suffering less severe stress in our experiment, as discussed in the preceding paragraph. It is possible that subordinate mice suffer more severe stress, resulting in alteration of adult neurogenesis and *Bdnf* expression, when they are kept under the group-housing condition for a longer period than was the case in the present study. In addition, it is possible that *Bdnf* expression in other brain areas, such as the ventral tegmental area, might play a critical role in behavioural change^16, 31^.

The present results suggest that the 5-HT system in the hippocampus behaves differently in dominant and subordinate males, and this may be associated with the behavioural differences between dominant and subordinate males. Some 5-HT receptors were more highly expressed in subordinate animals than in dominant animals, and the expression levels of these receptors were similar or lower in subordinate males compared with those of dominant males under fluoxetine treatment. Central 5-HT is synthesised in the midbrain raphe nuclei and plays various roles through at least 14 distinct receptor subtypes in different regions of the brain. Seven classes of 5-HT receptors are recognised, 5-HT_1–7_, of which all except 5-HT_3_ belong to the G-protein-coupled receptor superfamily^32^. The 5-HT_3_ receptor is a ligand-gated ion channel that belongs to the nicotinic acetylcholine receptor superfamily. The receptors are widely distributed throughout the brain and are expressed densely in the cortex, hypothalamus and limbic structures, including in the hippocampus, which is where the central regions affect aggression, anxiety- and depression-like behaviours. Serotonin plays different roles in such behaviours depending on the subtypes of the receptors involved in each specific behaviour. For example, the 5-HT_1A_ and 5-HT_2A_ receptors are coupled with the Gi/o and Gq proteins, respectively, and inhibit and excite, respectively, the innervation of target neurons. Knockout mice lacking these receptors therefore exhibit opposite behavioural responses in anxiety tests. The 5-HT_1A_ receptor knockout mice exhibit anxiogenic effects^33^, whereas 5-HT_2A_ receptor knockout mice exhibit anxiolytic effects^34^. It is therefore not easy to explain systematically the effects of the different degrees to which different 5-HT receptors were expressed in the current study. Social defeat stress causes 5-HT neuron activity to be suppressed through activation of GABA interneurons in the dorsal raphe nucleus^35^. It is therefore likely that up-regulation of 5-HT receptors in the hippocampus might be associated with differences in function of the 5-HT system. The finding that fluoxetine treatment eliminated the differential expression of these 5-HT receptors as well as anxiety- and depression-like behaviours between dominant and subordinate males supports this conclusion.

In the fluoxetine treatment experiments we treated all mice, including both dominants and subordinates, with the antidepressant. Given that the dominants and subordinates were determined after the start of the group-housing experiment, we were unable to treat only subordinates with fluoxetine. Further studies in which only subordinates are treated with the antidepressant, or in which the fluoxetine-treated and non-treated groups are compared in the same experimental session, are required to examine whether fluoxetine normalises the effect of subordination stress to the level of dominant animals.

Housing conditions, such as single housing versus group housing, have been observed to alter behaviour in mice^9^, but these results are controversial^10^. There are arguments pertaining to the effects of possible confounding factors, such as the time when mice are moved to single housing (e.g., pre-weaning or post-weaning or before or after sexual maturity is attained) and the number of mice kept in group housing (two mice or more)^19, 36^. In the current study, we did not compare the phenotype of mice kept in single-housing and group-housing conditions. It will be important to study how the phenotype of single-housed males is associated with the behaviours and gene expression levels observed in group-housed dominant and subordinate males.

The present results demonstrate that subordinate mice in naturally formed social hierarchies under group housing exhibit higher anxiety- and depression-like behaviour and altered gene expression levels compared with those of dominant mice. These results raise concern about the handling of experimental outcomes. Researchers often exclude injured individuals from experiments. The current findings indicate that this manipulation may shift experimental outcomes and emphasize the characteristics of dominant animals^8^. It is therefore desirable to establish animal housing conditions that minimise the differences between dominant and subordinate mice. Careful consideration of the housing condition after the mice attain sexual maturity may, depending on the nature of the experiments performed, be important to exclude uncontrollable biases caused by the dominance hierarchy.

## Methods

### Animals

Groups of six-week-old male C57BL/6NCrSlc (B6) mice, each group comprised of 32 mice to form eight group-housing cages, from a mixture of multiple litters were purchased from Japan SLC Inc. (Shizuoka, Japan) and kept in the animal facility of the National Institute of Genetics (NIG) in Japan. Group-housing experiments were performed using polycarbonate cages that were 18 cm wide, 17 cm high and 30 cm deep. The cages were produced for use in behaviour tests of “24 hour home-cage activity and social behaviour monitoring” (Ohara Co. Ltd., Tokyo, Japan). Four animals were kept in each cage, as described below. The animals were supplied with Palsoft paper bedding (Oriental Yeast, Tokyo, Japan). The animal room was maintained at a humidity of 50 ± 10% and a temperature of 23 ± 2 °C under a 12-h light/12-h dark cycle (the light period was from 06:00 to 18:00). The animals were allowed free access to CE-2 food (CLEA Japan, Tokyo, Japan) and water. Mice were maintained and all experiments were performed in accordance with NIG guidelines and all procedures were approved by the NIG Committee for Animal Care and Use (approval no. 26-9).

### Group housing of male mice in the home cage

The body weight of each animal was determined on the day the animal arrived at the NIG facility. Four animals of similar weights (mean difference between the largest and smallest individuals in a cage ± SEM was 1.04 ± 0.1 g) were housed together in each cage. The tails of the four males in each cage were marked with black permanent ink to allow each mouse to be recognised, then the animals were introduced into the cage. The bedding in each cage was changed every 3 or 4 days, and the tails of the mice were marked again when the bedding was changed. Each animal was weighed once weekly until the end of the experiment. After 2-weeks habituation, the dyadic agonistic interactions between each pair of animals in a cage were observed and the social ranks of the mice in the group determined (details given below) over the following 2 weeks (i.e., from when the mice were aged from 8 to 10 weeks old).

### Determination of the social hierarchy

The social behaviours of animals in a home cage were recorded using an infrared digital camera at the centre of the cage lid using “24 hour home-cage activity and social behaviour monitoring” apparatus (Ohara Co. Ltd.). The video data were saved using a commercially available DV-DH250S digital video recorder (Hitachi, Ltd., Tokyo, Japan). It has previously been observed that male mice exhibit agonistic behaviour during the early part of the dark phase^37^ or immediately after the cage has been cleaned, and that the social hierarchy in a group of male mice remains stable once established^17^. The recording was therefore automatically scheduled to run for 4 h (from 18:00 to 22:00) from immediately after the lights were switched off. In addition, video data were recorded for 2 h immediately after the cage had been cleaned during the light phase (twice weekly, usually between 10:00 and 14:00). Measurement of the frequency of dyadic agonistic interactions between each combination of two animals in the home cage was initiated after the mice in a cage had been co-housed for 2 weeks. The observation and definitions are shown in Table 1. Behaviour observation was conducted by a human observer using 4 h (dark phase) and 2 h (light phase) video recordings, but actual behavioural analysis was conducted for 20 min per day. When the initial agonistic interaction was observed, we started a 20-min observation window and conducted behavioural analysis for 20 min. Given the differences in time of initiation of agonistic interaction among cages, we set the observation window from the initiation of the first agonistic behaviour. Once male mice exhibited an agonistic interaction, they usually exhibited multiple agonistic interactions during the 20-min observation period. In order to observe agonistic interactions between different combinations of animals, we performed video observations for at least four different recording periods. Observations were made once every 3 or 4 days. If no agonistic behaviour was observed, a video from a different day was analysed. Observations were made for least four different days for each cage. Some cages were excluded from further analysis because animals did not show agonistic behaviour or exhibited a loop structure (e.g., mouse A was dominant to mouse B, mouse B was dominant to mouse C, but mouse C was dominant to mouse A).

### Behavioural tests

All animals in a cage were subjected to behavioural tests when the mice were between 10 and 12 weeks old to allow the relationships between social hierarchy and anxiety- and depression-like behaviour to be investigated. The behavioural tests were performed in the order open-field tests, elevated plus-maze tests, and forced swim tests (*n* = 16 cages for both Experiments 1 and 4). The behavioural tests were performed at least every other day between 07:00 and 09:00. The behavioural tests were conducted on all of the animals before the social hierarchy was characterised.

### Open-field test

Behaviour was observed for 10 min with a mouse in a square open field arena (60 × 60 cm) made of white polyvinylchloride plastic board with walls 40 cm high. The light level was 365 lux at the centre of the arena. The same procedure was used and the same observations made as described previously^38^. Each mouse was gently picked up by its tail with tweezers and placed in the same corner of the open field. During the ten-minute trial, their behaviour was recorded continually using a video camera placed over the centre of the arena, and the distance travelled and time spent in the centre of the arena were determined using a video tracking system (Image OF; Ohara Co. Ltd.) using National Institutes of Health ImageJ software. The frequencies and durations of other behaviours, such as rearing and stretching, were recorded with a resolution of 0.1 s using tanaMove software (version 0.01), which is freely available from the NIG website (http://www.nig.ac.jp/labs/MGRL/tanaMove.html)^39^.

### Elevated plus-maze test

The behaviour of a mouse was observed in an elevated plus maze made of white acrylic board with two open arms with short ledges (30 × 5 × 0.25 cm) and two closed arms (30 × 5 × 15 cm) enclosed within a clear acrylic wall extending from a central platform (5 × 5 cm). The maze was 60 cm above the floor and was dimly lit (150 lux). A mouse was placed in the central square and allowed to move freely for 10 min. The distance travelled (cm), time spent in an open arm, time spent in a closed arm, and time spent in the central square (all in seconds) were measured using a video tracking system (Image EPM; Ohara Co. Ltd.)^40^. The percentage of time spent in the open arms was calculated using the equation (time spent in the open arms/(sum of time spent in the open and closed arms) × 100).

### Forced swim test

The behaviour of a mouse in an acrylic container (12 × 22 cm) filled with water (15 cm deep, kept at room temperature) was recorded for 8 min, and the activity (mobility or immobility) of the mouse during the last 6 min was measured by observation using tanaMove software.

### Hormonal analysis

Corticosterone concentration was measured in blood collected between 07:00 and 09:00 by performing cardiopuncture after a mouse had been euthanised in CO_2_. The blood was centrifuged (at 3000 rpm for 30 min), and the serum was stored at −20 °C until ELISA analysis was performed. The corticosterone concentration can increase dramatically in response to handling stress, so all four mice in a cage (*n* = 16 cages) were euthanised at the same time, and the blood samples were collected concurrently by four well-trained technicians. Corticosterone is a physiological index of stress. The corticosterone concentration in each serum sample was determined using a rat corticosterone EIA kit (Enzo Life Science, Farmingdale, NY, USA). Each sample was analysed in duplicate. The intra- and inter-assay coefficients of variation were <10% and <7%, respectively.

### Gene expression analysis

#### RNA isolation and cDNA synthesis

Brains were collected from animals euthanised in CO_2_. The fresh brain was immediately removed from each euthanised animal (*n* = 8 cages for both Experiments 2 and 5). Blocks containing the hypothalamus and the hippocampus were cut from each brain, and the blocks were stored at −80 °C until gene expression analysis was performed. The hypothalamus and hippocampus tissues were homogenised in Invitrogen TRIzol^®^ Reagent (Thermo Fisher Scientific, Waltham, MA, USA) on ice. Total RNA was extracted, and the quantity and quality of the extracted RNA were checked using a Nano View Plus spectrophotometer (GE Healthcare Bio-Sciences, Pittsburgh, PA, USA). The purity of the RNA was assessed by determining the OD ratio (260/280 nm >2) and the 28S/18S rRNA ratio by denaturing RNA and separating the RNA in 1% agarose gel with ethidium bromide staining. DNase treatment (using a Ambion TURBO DNA-free™ kit; Thermo Fisher Scientific) was performed, then cDNA was synthesised from the RNA extracted from each brain region using the Primescript II 1st strand cDNA synthesis kit (TaKaRa Bio, Kyoto, Japan). All of the cDNA samples were stored at −20 °C until analysis by real-time PCR.

#### Real-time PCR analysis

The primers used are listed in Table S2. The primers for *Crh* (Mm01293920_s1), *Bdnf* (Mm04230607_s1) and *5-Htr1a* (Mm00434106_s1) were purchased from Applied Biosystems (Thermo Fisher Scientific). The primers for *Trkb* ^41^, *Creb1* ^42^, *Gr* ^43^, *5-Htr1b* ^42^, *5Htr2a* ^42^, *5Htr2c* ^42^, *5-Htr3a* ^42^, *5-Htr4* ^44^, *5-Htr7* ^42^ and *Gapdh* ^45^ were synthesised in accordance with the previous reports. The amount of each mRNA transcript was quantified using a TP800 Thermal Cycler Dice Real Time System (TaKaRa Bio) using SYBR Premix Ex Taq II Perfect Real Time (TaKaRa Bio). Each assay was performed in duplicate, and the amounts of the target genes detected relative to the amount of the reference gene (*Gapdh*) were calculated using the ΔΔCt method.

### Adult neurogenesis

#### Sample preparation

Newly formed cells were labelled by supplying the S-phase marker 5-bromo-20-deoxyuridine (BrdU; Sigma–Aldrich, St Louis, MO, USA) at a concentration of 1.2 mg/mL in drinking water for 14 days from when the mice were 10 weeks old until they were 12 weeks old (*n* = 8 cages). The animals were then deeply anaesthetised using sodium pentobarbital solution (100 mg/kg body weight; Ceva Sante Animale, Libourne, France) and perfused with 4% paraformaldehyde in 0.07 M phosphate buffer (pH 7.4). The brain was immediately removed, fixed overnight at 4 °C, then immersed in phosphate-buffered saline (PBS) containing 30% sucrose for 72 h at 4 °C. Each brain was then frozen in an optimal cutting temperature compound (Sakura Finetek, Torrance, CA, USA) using liquid nitrogen and kept at −80 °C. Each frozen brain containing the DG in the hippocampus was coronally sectioned into 30-µm sections in accordance with the mouse brain map (bregma coordinates −1.34 to −2.18 mm)^46^. The sections were mounted on slides and subjected to histological analysis.

#### Immunofluorescence and quantification

A total of eight sections per region per animal were analysed. Each section was incubated overnight at 4 °C with the primary antibodies, which were mouse anti-BrdU (1:50; G3G4; DSHB, Iowa, USA) and rabbit anti-NeuN (1:2000; Ab177487; Abcam, Cambridge, UK). The sections were washed with PBS and then incubated with the appropriate fluorescent donkey anti-rabbit secondary antibody Alexa488 (Cat. A-21206; Invitrogen) or donkey anti-mouse secondary antibody Alexa546 (Cat. A10036; Invitrogen) (ThermoFisher Scientific) for 90 min at room temperature. Sections were then counter-stained with DAPI solution (D9542; Sigma–Aldrich).

Immunofluorescence images were obtained using a confocal microscope (FV-1200; Olympus, Tokyo, Japan), and the numbers of BrdU/DAPI double-stained cells and BrdU/NeuN/DAPI triple-stained cells were bilaterally counted using image analysis software (FluoView; Version 10.0; Olympus). The length of the DG in each section was measured using NIH imageJ software, and the total number of positive cells in all sections was divided by this length and expressed as BrdU/DAPI and BrdU/NeuN/DAPI positive cells per unit of DG length^47^. BrdU/DAPI double-positive cells and BrdU/NeuN/DAPI triple-positive cells were defined as newly formed cells (proliferation) and newly formed neural cells (neurogenesis), respectively.

#### Fluoxetine treatment

Fluoxetine (0.8 mg/mL; Wako Pure Chemical Industries, Osaka, Japan) was supplied to group-housed male mice in drinking water from when the mice were 8-weeks-old until the end of the experiment. Fluoxetine-treated mice were subjected to the behavioural tests (*n* = 16 cages) described above and gene expression analysis of the hippocampus (*n* = 8) was performed as described above.

#### Data analysis

Two-way repeated measures ANOVA was used to examine the temporal change in body weight, followed by a *t*-test with Bonferroni correction as a *post-hoc* test. Most experimental datasets were not assumed to follow normal distributions, so we used non-parametric tests. Spearman’s rank correlation test was used to examine correlations between the data sets. The Wilcoxon-signed rank test was used to compare dominant and subordinate pairs of mice. Average data for each dominant and subordinate males in the same cage were used in pairwise statistical analyses. A difference was defined as statistically significant when the *P* value was <0.05. All statistical analyses were performed using the GraphPad Prism statistical analysis software (GraphPad Prism Software, La Jolla, CA, USA).

## Electronic supplementary material

Movie S1

Supplementary Information
