# Supplementary material for: Hierarchy in the home cage affects behaviour and gene expression in group-housed C57BL/6 male mice
Source: Sci Rep. 2017 Aug 1;7:6991. doi: 10.1038/s41598-017-07233-5 (PMC5539312; doi:10.1038/s41598-017-07233-5)
Supplement: Supplementary file 2 — Supplementary Information [file 41598_2017_7233_MOESM2_ESM.pdf]

Hierarchy in the home cage affects behaviour and gene expression in group-housed C57BL/6 male mice

Yasuyuki Horii, Tatsuhiro Nagasawa, Hiroyuki Sakakibara, Aki Takahashi, Akira Tanave, Yuki Matsumoto, Hiromichi Nagayama, Kazuto Yoshimi, Michiko T. Yasuda, Kayoko Shimoi, Tsuyoshi Koide

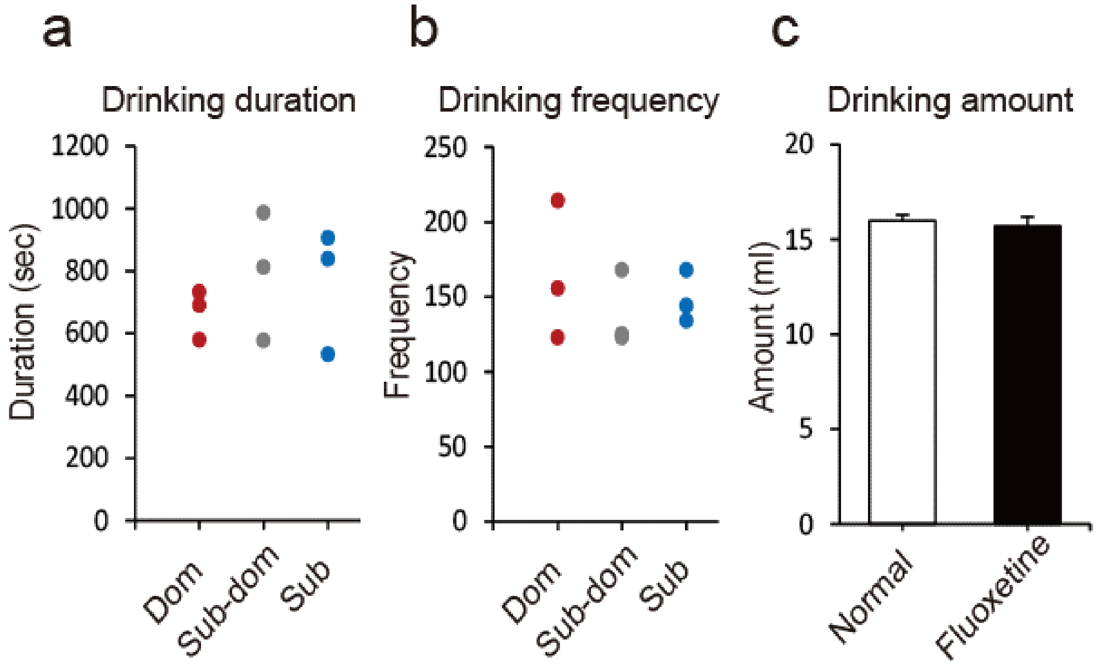

Figure S1. Comparison of fluid intake during fluoxetine treatment

(a and b) Drinking duration and frequency for group-housed mice with different social ranks (*n*=3 cages). We measured duration and frequency of fluid intake by video observation. Fluid intake was observed using 24-h videos recorded between 18:00 of day 12 to 18:00 of day 13 of fluoxetine treatment and during normal water treatment before the fluoxetine treatment. Drinking behaviour was detected when the mouth of the animal touched the nozzle of the water or fluoxetine fluid bottles. (c) Amount of normal water and fluoxetine-containing water consumed before and during the fluoxetine treatment, respectively (*n*=16 cages).

Table S1. Correlation between frequency of agonistic behaviour and gene expression in the brain

		Offensive behaviors		Defensive behaviors	
		Ag act	Sub act	Ag received	Sub react
Hypothalamus	*Crh*	#P=0.0607, r=-0.5557	#P=0.0568, r=-0.5627	#P=0.0520, r= 0.05719	n.s.
Hippocampus	*Bdnf*	*P=0.0142, r= 0.5990	n.s.	n.s.	n.s.
	Trkb	n.s.	n.s.	n.s.	n.s.
	Gr	n.s.	n.s.	n.s.	n.s.
	Creb1	#P=0.0502, r=-0.5135	*P=0.0102, r=-0.6401	n.s.	n.s.
	5-Htr1a	n.s.	n.s.	# P=0.0878, r= 0.4558	n.s.
	5-Htr1b	n.s.	n.s.	n.s.	n.s.
	5-Htr2a	n.s.	#P=0.0859, r=-0.4427	n.s.	n.s.
	5-Htr2c	n.s.	n.s.	n.s.	n.s.
	5-Htr3a	n.s.	n.s.	n.s.	n.s.
	5-Htr4	*P=0.0330, r=-0.5344	*P=0.0274, r=-0.5496	n.s.	*P=0.0314, r= 0.5386
	5-Htr7	n.s.	n.s.	n.s.	n.s.

Correlation between frequency of agonistic behaviour and gene expression level were examined by Spearman's rank correlation test. Cells shaded in green and red indicate the *P*-values were less than 0.1 and 0.05, respectively.

Table S2. Primer sequences used in the study

Gene	Forward primer	Reverse primer	Reference
Crh	Mm01293920_s1		purchased from Applied Biosystems
Bdnf	Mm04230607_s1		purchased from Applied Biosystems
Trkb	AAGGACTTTCATCGGGAAGCTG	TCGCCCTCCACACAGACAC	PMID: 21038447
Creb1	GGAATCTGGAGCAGACAACC	ATAACGCCATGGACCTGGAC	PMID:20002201
Gr	CAAAGCCGTTTCACTGTCC	ACAATTTCACACTGCCACC	PMID:12714642
5-Htr1a	Mm00434106_s1		purchased from Applied Biosystems
5-Htr1b	TATCACCTGTTGCACTGCTT	GAGATGGAGAAGACCCACAC	PMID:20002201
5-Htr2a	AGAACCCCATTCACCATAGC	ATCCTGTAGCCCGAAGACTG	PMID:20002201
5-Htr2c	GATTGGACTGAGGGACGAAA	ATGAAGAATGCCACGAAGGA	PMID:20002201
5-Htr3a	CTTCCCCTTTGATGTGCAG	CCACTCGCCCTGATTTATG	PMID:20002201
5-Htr4	TTCCCTGGACAGGTATTACGC	ATAGCCAAGCCAGAGGAAAG	PMID:16037544
5-Htr7	TTCTGCAACGTCTTCATCG	ATTCTGCCTCACGGGGTA	PMID:20002201
Gapdh	TGTGTCCGTCGTGGATCTGA	TTGCTGTTGAAGTCGCAGGAG	PMID:17140451
